# Impact of physician empathy on patient outcomes: a gender analysis

**DOI:** 10.3399/BJGP.2021.0193

**Published:** 2022-01-11

**Authors:** Caroline Surchat, Valerie Carrard, Jacques Gaume, Alexandre Berney, Carole Clair

**Affiliations:** University of Lausanne, Lausanne.; Psychiatric Liaison Service;; Department of Psychiatry — Addiction Medicine, Lausanne University Hospital and University of Lausanne, Lausanne.; Lausanne University Hospital and University of Lausanne, Lausanne.; Department of Training, Research and Innovation, Centre for Primary Care and Public Health (Unisanté), University of Lausanne, Lausanne.

**Keywords:** empathy, empathy measures, facial emotion recognition, general practice, patient-reported outcome measures, satisfaction, self report, gender, stereotypes

## Abstract

**Background:**

Empathy in primary care settings has been linked to improved health outcomes. However, the operationalisation of empathy differs between studies, and, to date, no study has concurrently compared affective, cognitive, and behavioural components of empathy regarding patient outcomes. Moreover, it is unclear how gender interacts with the studied dimensions.

**Aim:**

To examine the relationship between several empathy dimensions and patient-reported satisfaction, consultation’s quality, and patients’ trust in their physicians, and to determine whether this relationship is moderated by a physician’s gender.

**Design and setting:**

Analysis of the empathy of 61 primary care physicians in relation to 244 patient experience questionnaires in the French-speaking region of Switzerland.

**Method:**

Sixty-one physicians were video-recorded with two male and two female patients. Six different empathy measures were assessed: two self-reported measures, a facial recognition test, two external observational measures, and a Synchrony of Vocal Mean Fundamental Frequencies (SVMFF), measuring vocally coded emotional arousal. After the consultation, patients indicated their satisfaction with, trust in, and quality of the consultation.

**Results:**

Female physicians self-rated their empathic concern higher than their male counterparts did, whereas male physicians were more vocally synchronised (in terms of frequencies of speech) to their patients. SVMFF was the only significant predictor of all patient outcomes. Verbal empathy statements were linked to higher satisfaction when the physician was male.

**Conclusion:**

Gender differences were observed more often in self-reported measures of empathy than in external measures, indicating a probable social desirability bias. SVMFF significantly predicted all patient outcomes, and could be used as a cost-effective proxy for relational quality.

## INTRODUCTION

Empathy in primary care settings has been linked to improved health outcomes, such as patient satisfaction, adherence to treatment, and, by trickle effect, fewer malpractice complaints.^1^ However, there is as yet no consensus on the definition and operationalisation of empathy, making cross-study comparisons challenging.^2^

A comprehensive definition of empathy has been proposed by Decety and Jackson: *‘Feeling what another person is feeling, knowing what another person is feeling, and having the intention to respond compassionately to another person’s distress.’*^3^ This distinguishes affective, cognitive, and behavioural components of empathy. When it comes to the operationalisation of empathy, instruments used to measure these components can be classified into three categories: self-reported questionnaires (level of agreement with various empathy-oriented statements describing oneself), tests (performance tasks in which there is a correct empathic answer), and observational ratings (behaviours coded by external evaluators). Many studies have reported on the beneficial impact of physicians’ empathy;^4^^,^^5^ nevertheless, no study has concurrently compared these different measures in regard to patient outcomes. Different outcomes are expected, because self-reported empathy, tests, and observed empathy do not measure precisely the same construct of empathy.^6^ Moreover, self-reported measures are more prone to biases (for example, social desirability)^7^^–^^9^ than other measures.

Literature shows that empathy is highly influenced by gender. Stereotypically, females are considered more prosocial than males,^10^^,^^11^ and female physicians self-assess their empathy higher than male physicians do.^12^ Though females are expected to show more empathy,^13^ it is unclear whether gender differences can be observed across different types of empathy measures. If this difference is primarily driven by gender stereotypes, it is likely that more gender differences will be observed in self-reported questionnaires than in tests or external observations of empathy.^7^^,^^14^ On the contrary, if empathy is indeed more enacted by female physicians as a result of natural predisposition and/or social construct,^15^ gender differences will be observed in tests and external observations of empathy as well. Finally, patients may evaluate the display of empathy differently when standing in front of a male or female physician. Indeed, patients positively evaluate female physicians behaving in line with expected gender roles (softer voice, less dominance), whereas, for their male counterparts, a larger range of behaviour is related to patient satisfaction.^16^

The present project strives to fill in the literature gap regarding the concurrent analysis of different empathy dimensions with a gender perspective. The specific aims of this study are to investigate gender differences in six different empathy measures, compare these empathy measures regarding their relation to patient outcomes, and determine whether physicians’ gender impacts this relationship.

**Table:** How this fits in

The operationalisation of empathy differs between studies, and it is not known whether different empathy dimensions impact patient experience differently. This study examined the relationship between six empathy measures and patient satisfaction with, trust in, and quality of the consultation. As empathy is stereotypically viewed as a feminine quality, the gender of physicians was taken into account. This study pointed out the influence of stereotypes on self-reported empathy (with male physicians self-reporting lower empathic concern) but no gender difference in most of the behaviourally based empathy measures, and a significant link between Synchrony of Vocal Mean Fundamental Frequencies and patient outcomes.

## METHOD

### Study design and participants

The present study is a secondary analysis of data collected for a physician–patient communication study that received ethical approval from the regional ethic committees.^17^ More than 400 GPs in the French-speaking region of Switzerland were contacted to participate in a study on patient–physician communication. In total, 61 physicians (43% female) participated in the study. This represents a convenience sample. After being enrolled in the study, they filled in online questionnaires and took a test measuring their empathy and sociodemographic information.

Each participating physician was then video-recorded with the first two female and first two male patients agreeing to participate (recruited in the waiting room during a usual day of consultation), ending with 244 video-recorded consultations. Participating patients had to be aged >18 years, fluent in French, and present no documented psychiatric disorder. At the end of the consultation, patients indicated sociodemographic characteristics, as well as their satisfaction with the consultation, quality of the consultation, and their trust in the physician.

### Measures

This study compared six different measures of empathy measured through self-reported questionnaires, an online test, and external observation (Table 1).

**Table 1.** Measures of physician’s empathy: items, scales, missing, and Cronbach’s α

**Variables**
**Self-reported measures**

**Empathic concern**
Seven items: for example, ‘I am often quite touched by things that I see happen.’
Scale: 1 = ‘Does not describe me well’, 2 = ‘Rarely describes me well’, 3 = ‘Sometimes describes me well’,
4 = ‘Most of the time describes me well’, 5 = ‘Describes me very well’
Score: Mean of the seven items (after reversing specific reversed items) *n*= 58 physicians; missing values: *n* = 3 (4.9%), Cronbach’s α= .70

**Perspective taking**
Seven items: for example, ‘Before criticising somebody, I try to imagine how I would feel if I were in their place.’
Scale: 1 = ‘Does not describe me well’, 2 = ‘Rarely describes me well’, 3 = ‘Sometimes describes me well’,
4 = ‘Most of the time describes me well’, 5 = ‘Describes me very well’
Score: Mean of the seven items (after reversing specific reversed items) *n* = 58 physicians; missing values: *n* = 3 (4.9%), Cronbach’s α= .77

**Empathy online test**

**DANVA**
Participants are asked to determine which emotion is displayed in 24 portraits (happiness, sadness, anger,
or fear)
Scale: 0 = ‘False’, 1 = ‘Correct’
Score: sum of the number of emotions correctly recognised (0 to 24)
*N* = 58 physicians; missing values: *n* = 3 (4.9%), Cronbach’s α= .52

**External coding of empathy**

**VES with RIAS**
Aggregation of the statement frequencies of four categories (physician statements only): *empathy* (paraphrasing, interpreting, recognising, or naming other’s emotional state), *shows concern or worry* (indicates that a condition/event is serious, worrisome, distressing, or deserving special attention), *reassurance* (indicates optimism, encouragement, relief of worry, or reassurance), and *legitimise* (indicates that the other’s actions, emotions, or thoughts are understandable and normal)
Scale: number of statements per category divided by the total number of statements
Score: mean across the four categories
*n* = 243 sessions; missing values: *n* = 1 (0.4%)

**TES**
Nine items assessing affective, cognitive, and attitudinal aspects of the physician’s empathy such as concern for the patient, warmth, or understanding of the patient’s feelings.
Scale: 1 = ‘no display of empathy’, 7 = ‘extensive display of empathy’
Score: mean across the nine items
*n* = 241 sessions; missing values: *n* = 3 (1.2%)

**SVMFF**
Degree of synchrony of mean fundamental frequency of patient’s and physician’s voices
Estimates read as correlation coefficients [–1 to +1], positive estimates indicating higher synchrony.
*n* = 202 sessions; missing values: *n* = 40 ((19.6%).

*DANVA = Diagnostic Analysis of Nonverbal Accuracy. RIAS = Roter interaction analysis system. SVMFF = Synchrony of Vocal Mean Fundamental Frequencies. TES = Therapist Empathy Scale. VES = verbal empathy statements.*

#### Self-reported questionnaires of empathy

Physicians’ self-reported empathy was measured with two subscales of the Interpersonal Reactivity Index,^18^ known for its internal consistency.^19^ In the present study, the empathic concern subscale was used (which measured affective empathy), as was the perspective-taking subscale (which measured cognitive empathy).

#### Empathy test

Physicians filled in a validated emotion recognition test (the Diagnostic Analysis of Nonverbal Accuracy [DANVA])^20^ online. It consisted of 24 pictures of faces displaying one of four emotions (happiness, sadness, anger, or fear). Each picture was presented for 2 seconds, and the participant indicated which emotion was displayed. The final score was the number of emotions correctly recognised.

#### Observational empathy

Three external observational empathy assessments were included in the present study.

Verbal empathy statements (VES) were measured with the Roter interaction analysis system (RIAS),^21^ a validated coding system specifically designed for medical interactions. Certified coders classified the physician’s speech into 41 categories. To measure VES, a cluster used in previous studies in the field was applied.^22^ The number of statements for the categories ‘Empathy’, ‘Shows concern or worry’, ‘Reassures, encourages or shows optimism’, and ‘Legitimise’ (see Table 1 for more details) were aggregated and divided by the total number of intelligible statements.

Overall rating of physicians’ empathy was coded using the Therapist Empathy Scale (TES), a nine-item scale measuring behavioural display of empathy that showed internal consistency in past research.^23^

The Synchrony of Vocal Mean Fundamental Frequencies (SVMFF) has been proposed as a cost-effective alternative to the very time-consuming behavioural coding.^24^ This measure is based on the assumption that two individuals tend to synchronise their behaviour in highly empathic interactions,^24^^–^^27^ and thus are expected to synchronise their mean fundamental frequency (MFF), which relates to emotional arousal.^28^ Patients’ and physicians’ MFF was automatically measured every 0.25 seconds using Praat software version 5.3.82. The correlation between the patient’s and physician’s MFF was then computed across minutes while controlling for physician’s and patient’s gender (see Gaume *et al*^29^ and Baldwin *et al*^30^ for model details), ending with SVMFF scores ranging from −1 = total dyssynchrony (for example, patient displaying elevation of voice pitch while physician uses low pitch) to 1 = total synchrony.

### Patient outcomes

Patient outcomes were measured with three commonly used measures in healthcare studies: satisfaction, quality of consultation, and trust. These measures have been shown to relate to positive clinical outcomes such as less work impediment,^31^ better adherence to treatment,^32^^,^^33^ or higher quality of life,^34^ and were thus used as indicators of medical outcomes. Clinical outcomes were not measured as such. Satisfaction with the consultation was measured with the reversed single item: ‘I am not completely satisfied with my consultation with this doctor’. Quality of the consultation was assessed with the reversed single item: ‘Certain aspects of my consultation with this doctor could have been improved’. Both items originate from a validated scale^35^^,^^36^ and have shown good reliability in previous research.^37^^–^^39^ Finally, patients indicated their trust in the physician with the average (Cronbach’s α = .73) of four items (for example, ‘I completely trust my doctor’s decisions about which treatments are best for me’).

All outcome items were rated on a scale from 1 (do not at all agree) to 5 (completely agree). Because of the important ceiling effect (between 47% and 84% of the patients giving the maximum score), the outcome measures were dichotomised into two categories as follows: best score (5) versus any other score (1–4).

### Covariates

Four covariates were included: patient gender, frequency of consultations with this physician, years since first consultation with this physician, and physician clinical experience (aggregation of physician’s age, years since graduation, years of practice, and years since start of private practice; Cronbach’s α = .97).

### Statistical analysis

To investigate gender differences in the six empathy measures, separate independent sample *t*-tests were run comparing female and male physicians’ scores for each measure. Owing to skewness (indices between −0.94 and 0.94), nonparametric tests were also run, which showed similar results and are not presented in the result section.

To compare the different empathy measures regarding their relation to patient outcomes, and to determine whether the physician’s gender impacted this relationship, 18 logistic regression models were run (six empathy measures times three outcomes). Finally, these logistic regression models were replicated with an interaction term between physician’s gender and the empathy measure to test for gender effect on the relation between empathy and patient outcome. Each model controlled for the four covariates. Robust estimation was applied and the nested structure of the data (four patients nested in each physician) was accounted for with standard errors (SEs) adjusted for the clustering of the data. All analyses were performed using Stata (version 13.0).

## RESULTS

Male and female physicians did not significantly differ in terms of age and experience. However, they differed in the number of years since their beginning of private practice (average of 2.9 years later for females, adjusting for age), and in their working hours, with more females working part-time (Table 2). When it came to patients, males and females were similar in terms of age, education, severity of reason for consultation, and frequency of visits with this physician (Table 2). The patients participating in the present study had a slightly lower level of education on average, but similar age and health status compared with the general practice patients of other Swiss studies.^40^^–^^42^

*T*-tests analysing physician gender differences in empathy measures showed that most empathy measures (4/6) did not significantly differ between female and male physicians (Table 3). Nevertheless, female physicians self-rated their empathic concern significantly higher than male physicians did, and male physicians were significantly more vocally synchronised with their patient compared with female physicians.

**Table 2.** Descriptive statistics

**Physicians’ variables**	**Female physicians (*N*= 26)**					**Male physicians (*N*= 35)**					***t*-test**
		
	***n***	**Mean**	**SD**	**Min**	**Max**	***n***	**Mean**	**SD**	**Min**	**Max**	***t***
**Age**	25	50.5	9.1	33	70	33	51.6	8.1	39	65	0.99

**Years since graduation, *n***	25	23.5	9.1	3	42	33	25.8	8.2	13	40	1.98^d^

**Years since beginning medical practice, *n***	25	23.4	8.4	10	41	33	24.6	8.1	13	40	1.17

**Years since beginning private practice, *n***	25	12.5	8.8	1	33	33	16.6	10.1	2	34	3.29^e^

**Physician’s clinical experience (years)**	25	27.5	8.4	14.8	46.5	33	29.7	8.4	17	44.5	1.98^d^

**Working time (%)**	25	72.8	17.8	48	100	33	95.2	10.2	60	100	12.11^e^

**Patients’ satisfaction with consultation^a^**	104	4.7	0.8	1	5	140	4.8	0.7	1	5	0.30

**Patients’ evaluation of consultation quality^b^**	104	4.2	1.1	1	5	140	4.2	1.2	1	5	0.21

**Patient’s trust in physician^c^**	104	4.6	0.5	3.25	5	140	4.6	0.6	2.5	5	0.78

**Patients’ variables**	**Female patients (*N*= 122)**					**Male patients (*N* = 122)**					***t*-test**
		
	***n***	**Mean**	**SD**	**Min**	**Max**	***n***	**Mean**	**SD**	**Min**	**Max**	***t***
**Age**	122	57.3	18.5	18	97	122	57.7	17.5	19	91	0.18

**Years since first consultation with this physician**	122	9.4	9.3	0	43	121	9.3	9.7	0	44	0.07

**Patients’ satisfaction with consultation^a^**	122	4.8	0.6	2	5	122	4.7	0.9	1	5	1.56

**Patients’ evaluation of consultation quality^b^**	122	4.3	1.2	1	5	122	4.1	1.1	1	5	1.09

**Patient’s trust in physician^c^**	122	4.6	0.6	2.5	5	122	4.6	0.5	3.25	5	0.27

**Education**	***n***	**%**				***n***	**%**				***χ* ^2^**
Compulsory secondary education	33	27.1				22	18.0				4.68
Vocational training	51	41.8				58	47.5				
Tertiary education	23	18.9				23	18.9				
Advanced studies	14	11.5				15	12.3				
Bachelor’s degree	1	0.8				3	2.5				
Master’s degree	—	—				1	0.8				
Doctorate	—	—				—	—				

**Frequency of visits to this physician per year^f^**											
Less than once a year	19	15.7				18	14.8				1.37
Once or twice a year	30	24.8				35	28.7				
3 or 4 times a year	28	23.1				28	23.0				
5 or 6 times a year	11	9.1				14	11.5				
>6 times a year	33	27.3				27	22.1				

**Severity of reason for consultation**											
Not severe at all	59	48.4				48	39.3				9.83
Moderately severe	43	35.3				50	41.0				
Severe	18	14.8				15	12.3				
Very severe	1	0.8				8	6.6				
Extremely severe	1	0.8				1	0.8				

a *Scale: 1 = Very bad satisfaction, 2 = Bad satisfaction, 3 = OK satisfaction, 4 = Good satisfaction, 5 = Excellent satisfaction.*

b *Scale: 1 = Very bad quality, 2 = Bad quality, 3 = OK quality, 4 = Good quality, 5 = Excellent quality.*

c *Scale: 1 = Very bad trust, 2 = Bad trust, 3 = OK trust, 4 = Good trust, 5 = Excellent trust.*

d P<*0 .05.*

e P*<0 .001 .*

f *Data missing,* n *= 1. SD = standard deviation.*

**Table 3.** Independent sample *t*-tests for empathy measures between female and male physicians

**Variables**	**Female physicians**			**Male physicians**			***t*-test**			
	**Mean**	**SD**	**95% CI**	**M**	**SD**	**95% CI**	***t***	**DF**	***P*-value**	**Cohen’s *d***
**Empathic concern**	4.27	0.37	4.13 to 4.42	3.93	0.52	3.75 to 4.12	2.80	56	0.007^a^	.75
**Perspective taking**	3.78	0.53	3.56 to 3.99	3.72	0.63	3.50 to 3.96	0.30	56	0.763	.08
**DANVA**	18.08	2.70	16.99 to 19.17	18.06	2.53	17.15 to 18.97	0.02	56	0.983	.01
**VES**	0.69	0.48	0.60 to 0.78	0.66	0.50	0.58 to 0.75	0.44	241	0.659	.06
**TES**	3.43	0.77	3.28 to 3.58	3.25	0.76	3.12 to 3.38	1.84	239	0.067	.24
**SVMFF**	0.29	0.29	0.23 to 0.36	0.41	0.22	0.37 to 0.45	3.14	202	0.002^a^	.45

a P<*0.01. CI = confidence interval. DANVA = Diagnostic Analysis of Nonverbal Accuracy. DF = degrees of freedom. SD = standard deviation. SVMFF = Synchrony of Vocal Mean Fundamental Frequencies. TES = Therapist Empathy Scale. VES = verbal empathy statements.*

As shown in Table 4, the logistic regressions testing the relationship between the empathy measures and the patient outcomes showed that SVMFF was the only empathy measure related to patient outcomes. Additional logistic regression models with the interaction term between physician’s gender and empathy showed that the physician’s gender did not significantly impact the relation between empathy measures and patient outcomes, except for VES on patient satisfaction. In this model, a significant interaction was observed between VES and physician’s gender (*χ*^2^ = 18.28, *P*<0.05, odds ratio [OR] = 1.33, SE = 0.18, *P*<0.05). This result indicates that VES was linked to lower patient satisfaction when the physician was female, but to higher satisfaction when the physician was male.

**Table 4.** Logistic regression analysis of empathy dimensions predicting satisfaction, quality, and trust outcomes

	**Satisfaction**			**Quality**			**Trust**		
	**OR**	**SE**	**95% CI**	**OR**	**SE**	**95% CI**	**OR**	**SE**	**95% CI**
**Empathic concern^a^**	0.43	0.23	0.15 to 1.25	0.64	0.16	0.39 to 1.06	0.70	0.26	0.34 to 1.47
**Perspective taking^a^**	1.10	0.34	0.60 to 2.03	0.72	0.22	0.39 to 1.32	0.62	0.16	0.38 to 1.03
**DANVA^a^**	1.10	0.10	0.92 to 1.30	1.00	0.05	0.90 to 1.12	0.93	0.06	0.82 to 1.05
**VES^a^**	0.96	0.06	0.84 to 1.09	1.03	0.05	0.95 to 1.14	1.00	0.05	0.91 to 1.10
**TES^a^**	1.76	0.64	0.86 to 3.57	1.21	0.26	0.79 to 1.84	1.09	0.22	0.74 to 1.61
**SVMFF^a^**	4.59^b^	3.01	1.27 to 16.56	11.69^c^	7.85	3.14 to 43.56	3.61^b^	2.07	1.17 to 11.13

a *Each empathy measure was run in independent logistic regressions; ending with a total of six models for each outcome (that is, 18 models). Every model included the following covariates: frequency of consultations with this physician, time since the first consultation with this physician, an aggregate of highly correlated indicators of physician experience (physician’s age, number of years since graduation, number of years of practice, and year of the start of private practice), and the patient’s gender.*

b P<*0.05.*

c P<*0.001 CI = confidence interval. DANVA = Diagnostic Analysis of Nonverbal Accuracy. OR = odds ratio. SE = standard error. SVMFF = Synchrony of Vocal Mean Fundamental Frequencies. TES = Therapist Empathy Scale. VES = verbal empathy statements.*

## DISCUSSION

### Summary

This study aimed to compare six different empathy measures in relation to patient outcomes and physician gender. The study points out the influence of gender stereotype on self-reported empathy, with male physicians self-reporting lower empathic concern, but not differing from female physicians in most behaviourally based empathy measures. The divergent results between emotional concern and behavioural demonstration of empathy or emotion recognition tests could suggest that self-reported measures were influenced by gender stereotypes, that is, female physicians aligning their self-reported empathic concern with the stereotypical prosocial characteristics expected for their gender.^7^ Nevertheless, it is also possible that the number of opportunities to demonstrate empathy during these general practice consultations were too few, impeding the detection of any difference between female and male general physicians.

Synchrony measured with SVMFF showed a significant gender difference, with male physicians showing higher synchrony than their female counterparts. However, unlike the other empathy measures, synchrony was computed while considering both patient’s and physician’s behaviour. It may be the case that it was actually the patients who synchronised their vocal frequencies more when facing a male physician, and not the other way around. This could indicate that patients reacted to the status of power usually attributed to males (especially male physicians)^43^ by aligning their vocal frequency to them. More studies are needed to back up this hypothesis.

Counterintuitively, whereas numerous studies have underlined the beneficial impact of empathy on patients’ outcomes,^1^^,^^4^^,^^10^^,^^44^^–^^49^ this study revealed very few significant relationships between the empathy measures and patient outcomes, SVMFF being the only measure positively related to all outcomes. The setting of this study in primary care, with patients consulting for varied reasons (such as hypertension control or laboratory test feedback) may not have been the ground for an extensive demonstration of empathy. Thus, empathic display might have not been expected or acknowledged by the patients, explaining why empathy measures failed to predict outcomes. Moreover, synchrony may show different results compared with the other empathy measures, because it encompasses a broader concept than strictly empathy and could be considered as a proxy for relationship quality.

A higher count of VES was related to lower likelihood of patient satisfaction within consultations led by female physicians. This indicates that male physicians might be better rewarded than females when expressing their empathy. On the other hand, it is more surprising to observe that female physicians’ verbal empathy is related to less patient satisfaction. As other studies in the field suggest,^50^ female physicians’ verbal display of empathy might actually trigger more patient empowerment and enable them to feel more confident and dare to express more negative feedback, but more studies are needed to assess this.

### Strengths and limitations

The main strength of this study was to compare six measures of empathy covering the affective, cognitive, and behavioural components of empathy with outcomes. A variety of empathy measures was used (self-reported assessments, emotion recognition test, as well as external coding and a novel cost-effective proxy measure of empathy). However, VES and SVMFF encompass broader aspects of patient–physician communication than strictly empathy. In any case, the patient outcomes measured in the present study showed a typical high-ceiling effect, which lowered the variance that could be explained by the statistical models. Furthermore, the context of general practice might carry fewer or subtler opportunities for empathic display as compared with other settings such as psychiatry or oncology.^51^^–^^54^ Moreover, the sample of voluntary physicians, who tend to be interested in medical communication, have high interpersonal skills. This may have lowered the chances of revealing more important gender differences. Thus, the results of the present study may not be generalisable to the whole GP population.

### Comparison with existing literature

This study’s results showed that female physicians self-reported higher emotional concern than their male counterparts did, in line with existing literature regarding medical students^12^^,^^55^^,^^56^ and physicians.^12^ Similar results were reported in non-medical settings in youth^57^ and adults.^58^

Synchrony measured with SVMFF showed a significant gender difference, with male physicians showing higher synchrony than their female counterparts. Unfortunately, research on synchrony of voice frequency in clinical settings is rare, and studies focusing on other types of synchrony (facial mimicry, position, gesture, or lexical field alignment) report gender-aggregated data^24^^,^^59^ or use same-gender dyads,^26^^,^^60^^,^^61^ impeding any conclusions regarding gender-dyad differences.

SVMFF significantly predicted all patient outcomes. This result corroborates precedent studies showing that synchrony *‘embodies the patients’ self-reported quality of the relationship’*^26^ and is positively related to better medical outcomes,^62^ therapeutic alliance,^63^ and interpersonal trust.^64^

VES was only related to higher satisfaction within male-conducted consultations, in line with other studies reporting that male physicians seem to be better rewarded than females for their use of a patient-centred communication style,^65^^,^^66^ and that female physicians with better emotional recognition skills receive more ambivalent patient reactions than their male counterparts.^50^

### Implications for research

In the present study, self-reported empathy displayed more gender differences in comparison with other coded empathy. This result challenges the common notion that female physicians are more empathic than their male counterparts, and asks questions about the influence of gender stereotypes and gender expectations on empathy. Nevertheless, opportunities to demonstrate empathy may have been too rare in the present study’s setting, and more research should be conducted in fields where empathy is more central, such as in oncology, palliative care, or psychiatry. SVMFF significantly predicted patient outcomes, and could be used as a cost-effective proxy for relational quality in future studies. As SVMFF showed a significant gender difference, more gender studies of synchrony should be conducted in clinical settings to understand gender-dyad dynamics of synchrony.
